# Antioxidant and Cardioprotective Effects of EPA on Early Low-Severity Sepsis through UCP3 and SIRT3 Upholding of the Mitochondrial Redox Potential

**DOI:** 10.1155/2019/9710352

**Published:** 2019-08-26

**Authors:** Thibault Leger, Kasra Azarnoush, Amidou Traoré, Lucie Cassagnes, Jean-Paul Rigaudière, Chrystèle Jouve, Guilhem Pagès, Damien Bouvier, Vincent Sapin, Bruno Pereira, Jean-Marie Bonny, Luc Demaison

**Affiliations:** ^1^Université Clermont Auvergne, INRA, UNH, Unité de Nutrition Humaine, CRNH Auvergne, 63000 Clermont-Ferrand, France; ^2^Heart Surgery Department, Gabriel Montpied Hospital, Clermont-Ferrand University Hospital, Clermont-Ferrand, France; ^3^INRA, QuaPA UR370, F-63122 Saint-Genès-Champanelle, France; ^4^Université Clermont Auvergne, CHU Clermont-Ferrand, CNRS, SIGMA Clermont, Institut Pascal, Clermont-Ferrand, France; ^5^Department of Medical Biochemistry and Molecular Biology, CHU Clermont-Ferrand, Clermont-Ferrand, France; ^6^Department of Clinical Research and Innovation, CHU Clermont-Ferrand, Clermont-Ferrand, France

## Abstract

Sepsis still causes death, often through cardiac failure and mitochondrial dysfunction. Dietary *ω*3 polyunsaturated fatty acids are known to protect against cardiac dysfunction and sepsis lethality. This study set out to determine whether early low-severity sepsis alters the cardiac mitochondrial function in animals fed a Western-type diet and whether dietary eicosapentaenoic acid (EPA) administration protects the myocardium against the deleterious effects of sepsis and if so to seek possible mechanisms for its effects. Rats were divided into two groups fed either an *ω*3 PUFA-deficient diet (“Western diet,” DEF group) or an EPA-enriched diet (EPA group) for 5 weeks. Each group was subdivided into two subgroups: sham-operated rats and rats subjected to cecal ligation and puncture (CLP). *In vivo* cardiac mechanical function was examined, and mitochondria were harvested to determine their functional activity. Oxidative stress was evaluated together with several factors involved in the regulation of reactive oxygen species metabolism. Sepsis had little effect on cardiac mechanical function but strongly depressed mitochondrial function in the DEF group. Conversely, dietary EPA greatly protected the mitochondria through a decreased oxidative stress of the mitochondrial matrix. The latter was probably due to an increased uncoupling protein-3 expression, already seen in the sham-operated animals. CLP rats in the EPA group also displayed increased mitochondrial sirtuin-3 protein expression that could reinforce the upholding of oxidative phosphorylation. Dietary EPA preconditioned the heart against septic damage through several modifications that protect mitochondrial integrity. This preconditioning can explain the cardioprotective effect of dietary EPA during sepsis.

## 1. Introduction

Sepsis, due to bacterial contamination, affects 30 million people every year and is a leading cause of lethality worldwide, with some 6 million annual deaths [1]. Owing to poorer hygiene, the incidence of sepsis is higher in low- and middle-income countries, with a particular risk for people with vulnerable immune systems (neonates, elderly persons, pregnant women, hospitalized persons, and patients with AIDS, liver cirrhosis, cancer, kidney disease, diabetes, etc.). However, sepsis is also very frequent in industrialized countries and is a cost burden on their healthcare systems when it evolves toward septic shock [2].

Septic shock has been defined by Bone [3] as sepsis with persistent hypotension (systolic arterial pressure lower than 90 mmHg) despite adequate vascular filling, associated with abnormal tissue perfusion (lactic acidosis, oliguria, and disturbed consciousness). It is often observed in the course of resistance to antibiotic therapy and triggers life-threatening multiorgan dysfunctions. In 2010, approximately 15% of French patients diagnosed with sepsis developed septic shock, and even though the incidence of mortality has gradually decreased with new therapeutic advances, it remains high, with a rate of 39.5% [4]. Of the survivors, 42% of patients displayed at least one comorbidity. Importantly, cardiac failure accounts for approximately 50% of deaths [5], the heart being one of the preferred targets of the infection. However, except for a stimulation of the heart rate occurring in the early phase of sepsis [6], changes in cardiac mechanical function are detected only in the sickest patients using the most recent techniques (echography, MRI): they are first characterized by an impaired diastolic function, which evolves toward a reduced systolic function in the most severe cases [7, 8].

Therapeutic approaches must be applied soon after sepsis is triggered, with little knowledge of the subsequent disease severity. Determination of cardiac function is not an accurate way to estimate illness seriousness. It would be useful to have a cardiac parameter that is rapidly affected even in low-severity sepsis; a chosen therapy could then be appraised by how efficiently it upheld this parameter in physiological ranges.

The most recent hypothesis that offered to explain the cardiac disturbances encountered in sepsis involves bacterial lipopolysaccharides. These bind to Toll-like receptors which activate the NF-*κ*B pathway. NF-*κ*B migrates to the nucleus and triggers an acute inflammatory response. The cytokines released, mainly tumor necrosis factor-*α* (TNF-*α*) and interleukin-1*β* (IL-1*β*), depress mitochondrial function through a mechanism that involves severe oxidative stress and possibly calcium overload [9]. The oxidative stress inhibits energy production through suppression of the Krebs cycle aconitase [10] and reactivates the inflammatory response in a vicious cycle [11]. This leads to opening of the permeability transition pore, deenergization of the mitochondria, hypocontractility [12, 13], and cell death through apoptosis and/or necrosis [14, 15]. The mitochondria are definitely an important target in the etiology of sepsis [16].

Omega-3 (*ω*3) polyunsaturated fatty acids (PUFAs) are known to be cardioprotective in several pathophysiological situations including ischemia/reperfusion [17, 18], hypertrophy [19], and cardiac arrhythmias [20] through preservation of mitochondrial integrity [17, 18]. Eicosapentaenoic acid (C20:5 *n*-3 or EPA) is the best known of these [21, 22]. Omega-3 fatty acids are scant in Western diet, which is too rich in the proinflammatory *ω*6 PUFAs [23]. Animal studies using *ω*3 PUFA supplements clearly show that these fatty acids are protective during sepsis [24–26]. However, the mechanism of this protection is not yet properly documented and may involve the mitochondrial function.

This study set out to (i) determine whether the function of cardiac mitochondria is prematurely modified—before the occurrence of contractile dysfunctions—in early low-severity sepsis induced by cecal ligation and puncture in rats fed a Western diet, (ii) find out whether dietary eicosapentaenoic acid (C20:5 *n*-3 or EPA) protects the integrity of these organelles, and (iii) seek a potential mechanism responsible for observed effects. For this purpose, rats were fed either a diet deficient in *ω*3 PUFAs to model a typical Western diet or one enriched with EPA. Several features of the myocardial function were assessed: *in vivo* cardiac mechanical function, mitochondrial metabolism (rates of oxygen consumption, ATP production, and reactive oxygen release), fatty acid composition of cardiac phospholipids, and several parameters related to oxidative stress and inflammation. We hypothesized that (i) early low-severity sepsis causes increased inflammation and disruption of mitochondrial integrity in rats fed the Western-type diet and (ii) dietary EPA protects cardiac mitochondria through a decreased matrix oxidative stress related to increased UCP3 and SIRT3 protein expressions.

## 2. Materials and Methods

### 2.1. Ethical Approval

All the experiments followed the European Union recommendations for the care and use of laboratory animals for experimental and scientific purposes. All the animal work was approved by the local board of ethics for animal experimentation (Comité d'éthique pour l'expérimentation animale, Auvergne) and notified to the research animal facility of our laboratory (authorization no. APAFIS#2213-2016082409264678 v2). The research complied with the ARRIVE guidelines on animal research [27].

### 2.2. Experimental Rats and Diet

Forty 4-month-old female Wistar rats (Janvier, Le Genest-Saint-Isle, France) were maintained four per cage under controlled lighting, humidity, and temperature conditions. After one week of acclimatization, the rats were randomly allocated to two diet groups for 4 weeks. Each diet contained (in % *w*/*w*) casein 20, l-cysteine 0.3, lipid 5, starch 42.4, maltodextrin 12.7, sucrose 10, cellulose 5, a mineral mixture 3.5, a vitamin mixture 1, and choline 0.1. The lipid fraction contained approximately 2.25% of linoleic acid (C18:2 *ω*6 or LA). The fatty acid composition of the two diets is presented in Table 1. The first group of rats was fed a *ω*3 PUFA-deficient diet (DEF). In the second group, 0.625% of the monounsaturated fatty acids (MUFAs) was replaced by eicosapentaenoic acid (C20:5 *ω*3 or EPA) giving a *ω*6/*ω*3 PUFA ratio of 3.

### 2.3. Surgical Procedure

After the period of feeding, each diet group was subdivided into two equal subgroups. In the first subgroup, the cecum was externalized from the abdominal cavity and immediately repositioned (Sham subgroups); in the second, the cecum was ligated and punctured to induce sepsis (Sept subgroups). The cecal ligation and puncture (CLP) was performed according to Toscano et al. [28]. Briefly, the rats were anesthetized with isoflurane (induction 4%, maintenance 2%). After fur shaving, the external abdominal wall was disinfected with alcoholic betadine and a horizontal incision was made in the ventral wall at the level of the cecum to reach the abdominal cavity. The cecum was externalized and laid on the disinfected external abdominal wall. The cecum was ligated at 1 cm from the apex of the organ to trigger low-intensity sepsis. Two perforations were then made through the wall of the ligated cecum lining 0.5 cm apart on the upper face of the organ with a 20-gauge needle. Soft pressure was applied to the ligated part of the cecum to facilitate externalization of digesta on the outside of the organ. The cecum was reinserted immediately afterwards into the abdominal cavity, and buprenorphine (0.05 mg/kg of the body weight) was injected subcutaneously into the neck. The peritoneum was then closed with 6.0 silk sutures and the skin with sutures and metal clips. After the operation, anesthesia was stopped and the rats were placed one per cage in the animal facility where they woke 3–5 min after isoflurane withdrawal. The rats were then maintained in their cage for 48 h. The same diets as those given just before the surgical intervention were maintained during the postsurgery period.

To ensure that sepsis was induced, cardiac contractility was evaluated by MRI (see paragraph below). We observed that CLP tended to stimulate myocardial contractility (*p* = 0.08) similarly in both groups (cross-interaction between the effect of the diet and that of CLP = 0.99). This indicates clearly that animals of both groups were in the initial hyperdynamic phase of sepsis [6].

### 2.4. Cardiac MRI

At the end of the 48 h postsurgical period, *in vivo* cardiac function and morphology were assessed by nuclear magnetic resonance imaging (MRI). Anesthesia was induced in an anesthetic box using 5% isoflurane in a 30/70% air/oxygen mixture. The animals were then positioned prone in a rat cradle (Equipement Vétérinaire Minerve, Esternay, France) and maintained at 1.5–2% isoflurane at 1 liter/minute air/oxygen flow throughout the MRI experiments. The cradle was designed for positioning of the rat in the magnet. A tooth and ear bar secure system was installed along with lines for the delivery and scavenging of anesthetic gases, temperature control, and respiratory and electrocardiogram (ECG) monitoring. Temperature was maintained using a flow of heated air through the cradle. The ECG signal was not measured during cardiac imaging but was recorded in a few animals before electrode withdrawal and positioning in the magnet to estimate the heart rate for retrospective image reconstruction.

During cardiac MRI, high-resolution cine magnetic resonance imaging (cine-MRI) was carried out with a Bruker 117/16 BioSpec instrument equipped with an 11.7 T (500 MHz) horizontal magnet interfaced with an Avance III console running Paravision 6.0 software and a shielded BGA 9S gradient (Bruker BioSpin, Ettlingen, Germany). A 72 mm inner diameter quadrature volume coil (Bruker BioSpin, Ettlingen, Germany) was used for radiofrequency (RF) transmission and MRI signal reception. The MRI protocol for assessing myocardial function was modeled on the human short-axis left ventricular (LV) framework [29]. The navigator-based self-gated fast low-angle shot (IntraGate FLASH) bright blood sequence was used for cine imaging of the heart. Ten to 12 contiguous 1 mm slices were acquired to cover the heart from the base to the apex.

For the image analysis, the navigator slice was positioned parallel to the image slice, centered to the heart and crossing the lungs to maximize its sensitivity to cardiorespiratory movements. The imaging parameters were as follows: repetition time (TR)/echo time (TE) 6/2.5 ms, flip angle (FA) 8 degrees, field of view (FOV) 5 × 5 cm, matrix 256 × 256, and slice thickness 1 mm. Four hundred repetitions were performed during acquisition of the whole raw dataset, and 16 movie frames were reconstructed using the Bruker IntraGate reconstruction algorithm, which was fed with the heart rate value (~400 bpm) recorded prior to the MRI and the mean animal respiratory frequency as estimated parameters.

Image analysis was performed using the free Segment v2.1 R5960 software [30]. Images were first converted into DICOM format before loading into Segment software for segmentation using the built-in “alternative left ventricular tool” algorithm. For each rat, end-diastolic volume (EDV) and end-systolic volume (ESV) were first identified by the global maximum and minimum left ventricle (LV) cavity volumes, respectively, to make sure that all the slices were in phase. Semiautomatic segmentation of the LV was then performed as described elsewhere [31]. Briefly, a point was first seeded in the center of the LV blood pool, from which the algorithm fits a deformable model that expands toward the endocardial border to include the LV blood pool across the various short-axis slices. Manual adjustments were performed as required afterward mainly for the epicardial border in general and for the endocardial border where the papillary muscles appear separated from the myocardium. Stroke volume (SV = EDV − ESV), ejection fraction (EF = SV/EDV), and cardiac output (CO = SV × heart rate) were calculated from the end-diastolic and end-systolic volumes. The EDV time curve was then used to derive the contraction and relaxation rates.

### 2.5. Preparation of Isolated Mitochondria

At the end of the cardiac MRI, about 200 mg of the apical heart was freeze clamped in liquid nitrogen and stored at −80°C for later analysis. The mitochondria were extracted as previously described [17]. Atria and the remaining aorta were cut off from the heart. The myocardium was shredded with scissors in a cold isolation buffer A composed of mannitol 220 mM, sucrose 70 mM, MOPS 5 mM, and EGTA 2 mM, pH 7.4 at 4°C, fatty acid-free serum albumin 0.2%. The shreds of the myocardium were rinsed several times on a filter and homogenized with a Polytron blender (0.4 s, rheostat = 3) and a Potter-Elvehjem tissue grinder containing 15 ml of isolation buffer A. After centrifuging (800 × *g*, 10 min, 4°C), the supernatant with subsarcolemmal mitochondria was kept in ice. The pellet was resuspended in a cold isolation buffer B composed of KCl 100 mM, MOPS 50 mM, and EGTA 2 mM, pH 7.4 at 4°C, fatty acid-free serum albumin 0.2%. A protease (subtilisin 0.02%) was added for 1 min to digest myofibrils at ice temperature, and the whole was then homogenized with a tissue grinder (300 rpm, 3 to 4 transitions). Immediately afterward, subtilisin action was stopped by adding isolation buffer (30 ml) and centrifuging (8000 × *g*, 10 min, 4°C). The supernatant was discarded and the pellet was resuspended in isolation buffer B. The homogenate was then centrifuged (800 × *g*, 10 min, 4°C), and the resulting supernatant with intermyofibrillar mitochondria was collected and filtered with the other supernatant. Mitochondria were then washed by centrifuging (8000 × *g*, 10 min, 4°C). The pellet of mitochondria was suspended in cold isolation buffer C composed of KCl 100 mM, MOPS 50 mM, and EGTA 1 mM, pH 7.4 at 4°C, recentrifuged (8000 × *g*, 10 min, 4°C), and finally resuspended at a concentration of approx. 15 mg/ml.

### 2.6. Respiration Measurements

The rate of mitochondrial oxygen consumption was measured at 30°C in an incubation chamber (Oxytherm OXYT1, Hansatech, King's Lynn, England) with a Clark-type O_2_ electrode filled with 1 ml of incubation medium (KCl 125 mM, Tris-HCl 20 mM, KH_2_PO_4_ 10 mM, and EGTA 1 mM, pH 7.2 at 30°C, fatty acid-free bovine serum albumin 0.15%). All measurements were made using mitochondria (0.2 mg of mitochondrial protein/ml) incubated with either glutamate (5.5 mM)-malate (2.5 mM), palmitoylcarnitine (20 *μ*M)-malate (2.5 mM), or succinate (5.5 mM)-rotenone (2 *μ*M) as substrates (state 2), in the presence of ADP 100 mM (state 3) and afterwards with the addition of oligomycin 0.5 *μ*g/ml (state 4). The pH of each substrate was strictly fixed at 7.4 at 30°C. The incubation medium was maintained at 30°C and constantly stirred with a built-in electromagnetic stirrer and bar flea. Coupling of the oxidative phosphorylation was assessed by the state 3 to state 4 respiration rate ratio, which measures the degree of control exerted on oxidation by phosphorylation (respiratory control ratio or RCR). Amplex® Red (1 *μ*M) and horseradish peroxidase (5 U/ml) were added to the medium at the beginning of the respiration measurement to maintain the same experimental conditions as those used in the measurement of H_2_O_2_ release.

### 2.7. Mitochondrial Reactive Oxygen Species Release

The rate of mitochondrial reactive oxygen species release was evaluated in the same conditions as the respiration measurements. It followed the linear increase in fluorescence (excitation at 560 nm and emission at 584 nm) due to enzymatic oxidation of Amplex® Red by H_2_O_2_ in the presence of horseradish peroxidase modified to kinetically follow the rate of production of reactive oxygen species by isolated mitochondria on a fluorometer (Xenius XC, SAFAS, Monaco). Reaction conditions were 0.20 mg of mitochondrial protein/ml, 5 U/ml of horseradish peroxidase, and 1 *μ*M of Amplex® Red with the same substrates and concentrations as in the respiration measurements.

### 2.8. Gene Expression

Total RNA was extracted from 50 mg of heart powder using TRIzol® (Thermo Scientific) according to the manufacturer's instructions. Chloroform was added (0.2 ml/ml of TRIzol®), and samples were mixed and centrifuged for 15 min at 12000 × *g* at 4°C. An aqueous phase containing ribonucleic acid (RNA) was collected, mixed with isopropanol to precipitate RNA, and centrifuged (12000 × *g*, 4°C, 15 min). After centrifuging, the pellet was washed with ethanol 70% (vol/vol), dried, and suspended in water. RNA quantification and integrity were verified by measuring the ratio of optical densities at 260 nm and 280 nm and by agarose gel migration, respectively. cDNAs were synthesized from 2 *μ*g of total RNA using the High-Capacity cDNA Reverse Transcription Kit from Applied Biosystems (Thermo Fisher Scientific, Asnières-sur-Seine, France). The products of reverse transcription were used for quantitative real-time polymerase chain reaction (qRT-PCR) using specific primers and Rotor-Gene SYBR Green PCR master mix on a Rotor-Gene Q system (QIAGEN, Courtaboeuf, France). Messenger RNA (mRNA) was quantified using the standard curve of native cDNA and serial dilutions. The cardiac mRNA expressions were determined for hypoxia-inducible factor-1 (HIF-1), sirtuin-3 (SIRT3), cytosolic Cu-Zn superoxide dismutase (SOD1), mitochondrial manganese-dependent superoxide dismutase (SOD2), interleukin-1*β* (IL-1*β*), tumor necrosis factor-*α* (TNF-*α*), hexokinase 2 (HK2), phosphofructokinase-2 (PFK2), pyruvate kinase muscle-type (PKm), M and H lactate dehydrogenases (LDH M and H), and pyruvate dehydrogenase kinase 4 (PDK4). Primer sequences and PCR conditions are presented in Table 2. Some housekeeping genes were tested (*β*-actin and hypoxanthine phosphoribosyltransferase); *β*-actin was finally chosen for its intergroup stability.

### 2.9. Western Blot Analysis

Tissues were ground three times in a mini bead beater in the presence of a lysis buffer made of HEPES 50 mM, sodium chloride 150 mM, EDTA 10 mM, anhydrous sodium tetrabasic pyrophosphate 10 mM, *β*-glycerophosphate 25 mM, sodium fluoride 100 mM, and anhydrous glycerol 1.086 M supplemented with phosphatase inhibitors (Sigma Aldrich, Saint-Quentin-Fallavier, France). Successive centrifugations were performed and the supernatants were collected. Proteins were quantified using a bicinchoninic acid assay kit (Thermo Fisher Scientific, Asnières-sur-Seine, France). For protein immunoblotting, 25 *μ*g of proteins was loaded for separation by SDS-PAGE electrophoresis and transferred on PVDF membranes. Membranes were then immunoblotted with the appropriate antibody to detect the nuclear factor of the kappa light polypeptide gene enhancer in B-cell inhibitor alpha (I*κ*B*α*) (1 : 1000, Cell Signaling #9242), voltage-dependent anion-selective channel (VDAC) (1 : 1000, Cell Signaling #4866), sirtuin-3 (SIRT3) (1 : 500, Santa Cruz #sc-365175), uncoupling protein-3 (UCP3) (1 : 200, Santa Cruz #sc-31387), nitrosylated tyrosine (1 : 1000, Thermo Scientific #32-1900), acetylated lysine (1 : 1000, Cell Signaling #9441), and peroxisome proliferator-activated receptor gamma coactivator 1-alpha (PGC-1*α*) (1 : 5000, Santa Cruz #sc-13067). Antibody binding was detected using HRP-conjugated secondary antibodies and ECL Western blotting substrate (Thermo Fisher Scientific, Asnières-sur-Seine, France). Immunoblots were visualized using a chemiluminescence imaging system (MF-ChemiBIS, DNR Bio-Imaging Systems, Jerusalem, Israel) and quantified using Multi Gauge V3.2 software. For detection of nitrosylated proteins, protein extraction was performed according to the manufacturer's instructions. Several housekeeping proteins including *α*-tubulin (1 : 1000, Sigma #8203) and GAPDH (0.2 *μ*g/ml, Sigma #G9545) were tested, but none was stable across the four subgroups. However, protein densitometry to Ponceau red was stable. It was thus decided to normalize all the relevant proteins with this parameter.

### 2.10. Lipid Analysis

Total lipids were extracted from cardiac tissues according to Folch et al. [32]. Lipid extracts (approx. 50 mg) were prepared using 4 ml chloroform-methanol (2 : 1 *v*/*v*) and 1 ml 0.9% NaCl. After phospholipid separation on Sep-Pak cartridges, fatty acid methyl esters were prepared and separated by gas chromatography as previously described [33].

### 2.11. Cardiac Oxidative Stress

Mitochondrial SOD was assayed using a commercially available kit (Bertin Pharma, Montigny-le-Bretonneux, France).

### 2.12. Statistical Analysis

Results are presented as mean ± SEM. The data underwent a two-way analysis of variance (ANOVA) describing the effects of the diets and of sepsis and the cross-interaction between them. When necessary, the means were compared using Fisher's least significant difference (LSD) test. A probability lower than 0.05 was considered significant. Statistical analysis was performed using the NCSS 2007 software (NCSS LLC, Kaysville, UT).

## 3. Results

### 3.1. General Data

The body weights of the animals before surgery were similar in the four subgroups (243 ± 5, 245 ± 7, 266 ± 7, and 250 ± 7 g for the DEF Sham, DEF Sept, EPA Sham, and EPA Sept, respectively). The surgery tended to decrease these variables, but the observed changes were not significant (data not shown). Since a large part of the myocardium was used for mitochondrial preparation, the heart weights of these rats were not determined.

### 3.2. Fatty Acid Composition of Cardiac Phospholipids

The fatty acid composition of membrane phospholipids plays a crucial role in the regulation of cardiac and mitochondrial function because of not only its involvement in prostanoid synthesis but also its engagement in the modulation of enzymatic activities through changes in membrane fluidity.

The EPA-rich diet did not notably modify the proportions of saturated fats in cardiac membranes (Table 3), but it decreased those of the monounsaturated fatty acid C18:1*ω*9 (−22% and −16% in the Sham and Sept subgroups, *p* < 0.001). Although the proportions of total PUFAs were unchanged, their profile was considerably altered by the diets. PUFAs of the *ω*6 series were reduced by the EPA-rich diet (−19% and −22% for the Sham and Sept subgroups, *p* < 0.001), and those of the *ω*3 family were correspondingly increased (+124 and +145%, *p* < 0.001). Arachidonic acid (ArA or C20:4*ω*6) and its first metabolites (C22:4*ω*6 and C22:5*ω*6) were thus decreased, and EPA, C22:5*ω*3, and C22:6*ω*3 were greatly increased (*p* < 0.001 for all the comparisons).

Sepsis triggered certain modifications of the phospholipid fatty acid profile that affected only PUFAs. In the DEF group, the infectious disease reduced the first metabolites of ArA (−10% and −10% for C22:4*ω*6 and C22:5*ω*6, *p* < 0.05). In the EPA group, the pathology increased the proportion of total *ω*3 PUFAs, mainly C22:6*ω*3 (+11 and +13%, *p* < 0.05).

### 3.3. Sepsis-Induced Myocardial Inflammation

**Figure 1:**
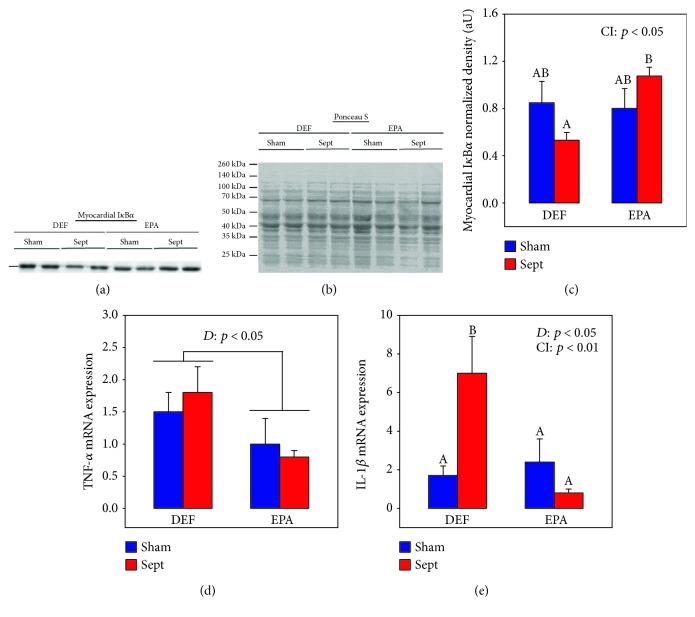
Cardiac inflammation parameters: (a) representative immunoblots of myocardial I*κ*B*α*; (b) protein densitometry to Ponceau S of the corresponding samples; (c) quantification of myocardial I*κ*B*α*; (d) TNF-*α* mRNA expression; (e) IL-1*β* mRNA expression. The means are a consortium of 8 rats 48 h after the surgery. DEF: rats fed the *ω*3 polyunsaturated fatty acid-deficient diet; EPA: rats fed the eicosapentaenoic acid-enriched diet; Sham: sham-operated rats; Sept: septic rats; *D*: diet effect; CI: cross-interaction between the effect of the diet and that of sepsis; A, B: histogram bars on the same panel without a common letter are significantly different.

Bacterial lipopolysaccharides link to Toll-like receptors and activate the NF-*κ*B pathway. This activation is characterized by the release and degradation of I*κ*B*α* as well as migration of the NF-*κ*B moiety to the nucleus and induction of the proinflammatory cytokine expression. The reduction of I*κ*B*α* was determined in the myocardial tissues of the four subgroups of animals (Figure 1(c)). Dietary EPA did not alter its value in sham-operated animals but increased it after sepsis (+103%, *p* < 0.001) compared to Sept animals fed the DEF diet.

Figure 1 also depicts the effects of dietary EPA and sepsis on cardiac TNF-*α* and IL-1*β* mRNA expressions. Gene expression of TNF-*α* (Figure 1(d)) was not affected by sepsis but depended on the dietary PUFA ingested: it was significantly decreased by dietary EPA (−33% and −56% in the Sham and Sept subgroups, *p* < 0.05 in general). IL-1*β* mRNA expression (Figure 1(e)) behaved differently: it was considerably increased by CLP in the DEF group (+530%, *p* < 0.01), but not at all in the EPA group (−67%, NS, CI: *p* < 0.01).

### 3.4. MRI Study

The *in vivo* cardiac morphology and function were determined by MRI (Table 4) to know whether these parameters were sensitive enough to the effects of the early low-severity sepsis and EPA treatment. Cardiac morphology estimated by the end-diastolic and end-systolic volumes was minimally affected by the diets and surgery. This was also the case for the cardiac function parameters, including ejection fraction, heart rate, cardiac output, and relaxation. However, an interesting tendency was noted for contraction: sepsis tended to increase the contraction rate in both diet groups (+14% and +13% for the DEF and EPA groups, *p* = 0.085): no diet effect was noted, however.

### 3.5. Mitochondrial Function

Cardiac mitochondria were immediately harvested after sacrifice to evaluate their energy metabolism and reactive oxygen species release (Table 5) to determine whether the function of these organelles was modified by sepsis and dietary EPA. The main modifications were observed when glutamate/malate was used as substrate. In the DEF groups, sepsis reduced the state 3 respiration rate (−42%, *p* < 0.01), respiratory control ratio (RCR) (−31%, *p* < 0.01), and rate of ATP production (−37%, *p* < 0.05). Although the basal rates of H_2_O_2_ release during states 3 and 4 of the respiration were unchanged, the ratios of H_2_O_2_ release to the state 3 respiration rate and of H_2_O_2_ release to the rate of ATP production were greatly increased by sepsis (+70 and +128%, respectively, *p* < 0.05). Interestingly, none of these parameters (state 3 respiration rate, RCR, H_2_O_2_ release/state 3 respiration rate, and H_2_O_2_ release/rate of ATP production) were altered by sepsis in the EPA-fed rats.

When succinate/rotenone or palmitoylcarnitine/malate was used as substrates, the same trends were observed for the rates of state 3 respiration and ATP production but the differences were not significant. However, for the H_2_O_2_ release normalized to the rates of state 3 respiration or ATP production, the differences generally remained significant like those observed with glutamate/malate as substrates.

### 3.6. Glycolytic Enzyme mRNA Expressions

**Figure 2:**
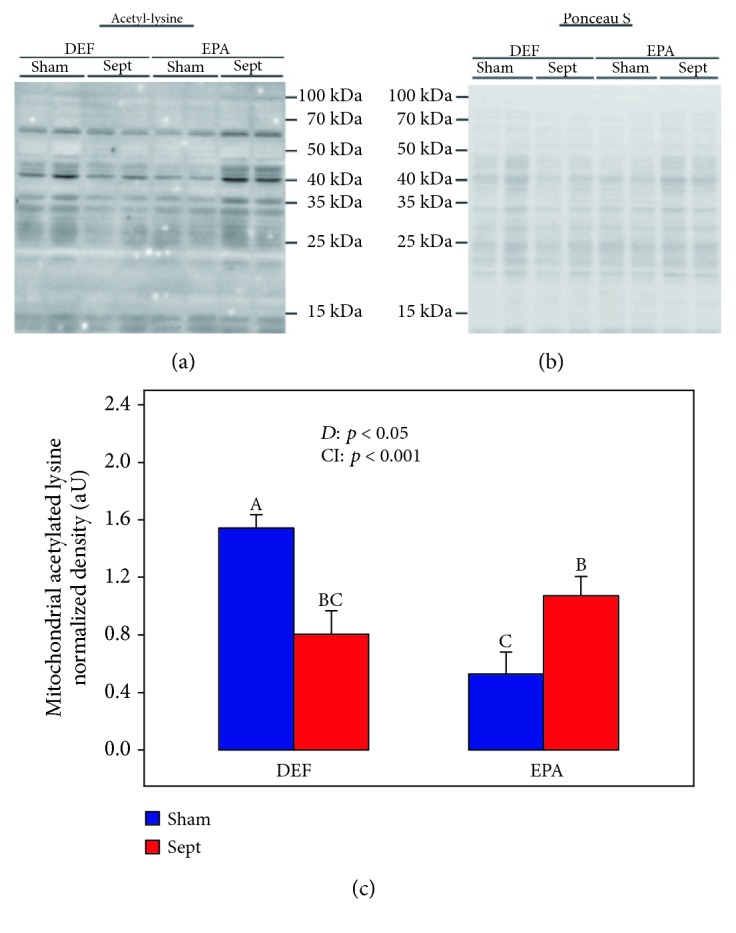
Degree of acetylation of mitochondrial proteins: (a) representative immunoblots; (b) protein densitometry to Ponceau S of the corresponding samples; (c) quantification of protein acetylation. The means are the average of 8 rats. DEF: rats fed the *ω*3 polyunsaturated fatty acid-deficient diet; EPA: rats fed the eicosapentaenoic acid-enriched diet; Sham: sham-operated rats; Sept: septic rats; *D*: diet effect; CI: cross-interaction; A, B, C: histogram bars on the same panel without a common letter are significantly different.

High glucose oxidation and low fatty acid degradation are often associated with a cardioprotective metabolic profile [34, 35].  Table 6 summarizes the mRNA expression of several glycolytic enzymes in order to estimate glucose metabolism. Sham-operated rats fed the different diets had the same glycolytic enzyme mRNA expressions except for that of pyruvate kinase, which was reduced by the EPA diet (−30%, *p* < 0.05 by one-way analysis of variance). Pyruvate dehydrogenase kinase 4 gene expression was also decreased by the EPA diet (−62%, *p* < 0.05 by one-way analysis of variance). Sepsis had little effect on the glycolytic enzyme mRNA expression in the DEF diet-fed rats: it tended only to increase the hexokinase 2 gene expression (+41%, *p* = 0.058 by one-way analysis of variance). Conversely, it strongly decreased the expression of several enzymes in the EPA diet-fed rats: phosphofructokinase 2 (−41%, *p* < 0.05), pyruvate kinase (−61%, *p* < 0.01), and muscle (−37%, *p* < 0.01) and cardiac (−60%, *p* < 0.01) lactate dehydrogenases. The hexokinase 2 and pyruvate dehydrogenase kinase 4 mRNA expressions were also decreased (−50% and 77%, respectively, *p* < 0.01 by one-way analysis of variance). Mitochondrial acetylated lysine reflects the rate of *β*-oxidation [36]. In sham-operated animals, acetylated lysine expression (Figure 2) was decreased by dietary EPA (−66%, *p* < 0.001). Sepsis reduced lysine acetylation in the DEF group (−48%, *p* < 0.01) but increased it in the EPA group (+102%, *p* < 0.05).

### 3.7. Oxidative and Nitrosative Stresses

**Figure 3:**
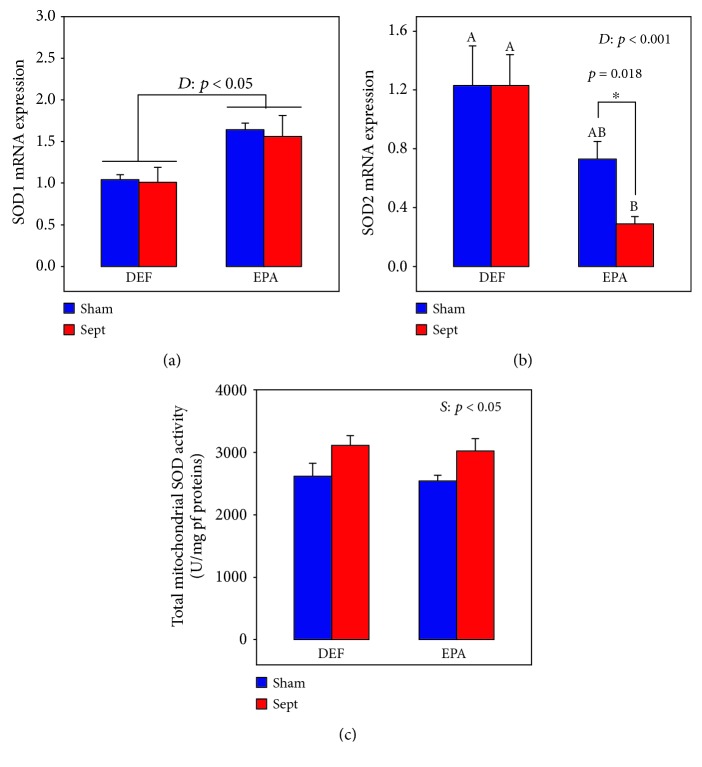
Cytoplasmic (SOD1) (a) and mitochondrial (SOD2) (b) superoxide dismutase mRNA expressions and mitochondrial SOD activity (c) in female rats subjected to a 48 h sepsis. The means are the average of 8 rats. DEF: rats fed the *ω*3 polyunsaturated fatty acid-deficient diet; EPA: rats fed the eicosapentaenoic acid-enriched diet; Sham: sham-operated rats; Sept: septic rats; *D*: diet effect; *S*: sepsis effect; A, B: histogram bars on the same panel without a common letter are significantly different; ^∗^significantly different by one-way analysis of variance.

The oxidative stress was further evaluated in the whole myocardium by determining SOD mRNA expressions. The cytosolic SOD1 (Figure 3(a)) was increased in the EPA subgroups (+58 and +54% for the Sham and Sept subgroups, *p* < 0.05 in general) compared with the DEF subgroups but was not affected by sepsis. By contrast, the gene expression of the mitochondrial SOD 2 (Figure 3(b)) was reduced by the EPA-rich diet (−59%, *p* < 0.001) but this was significant only in the Sept subgroup (−76%, *p* < 0.001). This parameter was not affected by sepsis in the DEF group. The total mitochondrial SOD activity (Figure 3(c)) was not influenced by the diets but was increased by sepsis (+19% for the DEF plus EPA groups, *p* < 0.05).

**Figure 4:**
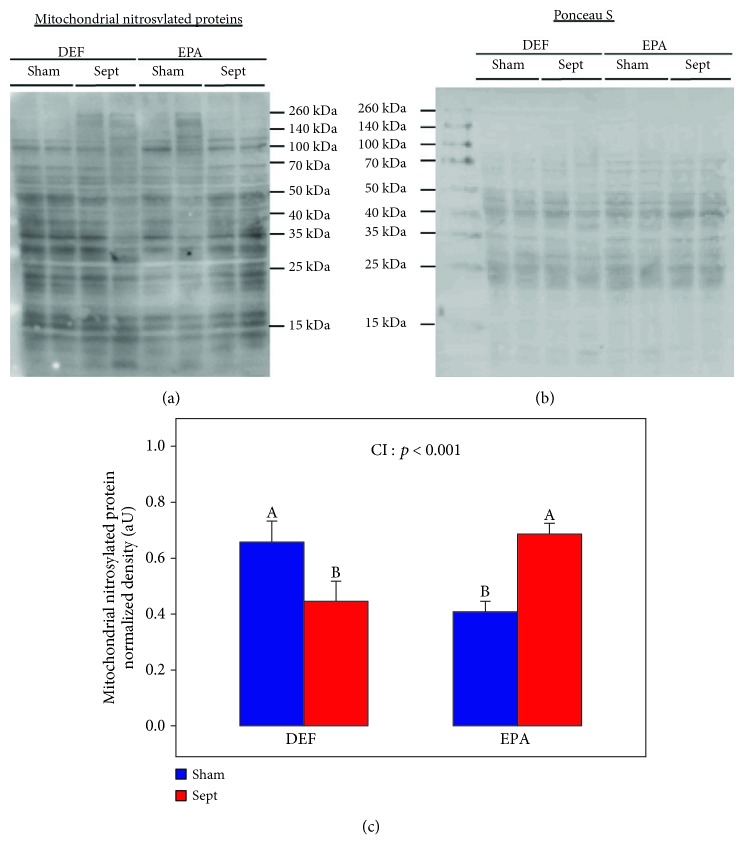
Degree of mitochondrial nitrosylated proteins: (a) representative immunoblots; (b) protein densitometry to Ponceau S of the corresponding samples; (c) quantification of mitochondrial nitrosylated proteins. The means are the average of 8 rats. DEF: rats fed the *ω*3 polyunsaturated fatty acid-deficient diet; EPA: rats fed the eicosapentaenoic acid-enriched diet; Sham: sham-operated rats; Sept: septic rats; CI: cross-interaction; A, B: histogram bars without a common letter are significantly different.

Nitrosylation intensity of mitochondrial proteins is presented in Figure 4. Sham-operated animals of the EPA group displayed a lower degree of nitrosylation (−55%, *p* < 0.05) than their counterparts fed the DEF diet. Sepsis reduced this parameter in the DEF group (−26%) but increased it in the EPA group (+51%, CI: *p* < 0.001). Finally, septic rats in the EPA diet group displayed a higher degree of mitochondrial protein nitrosylation (+54%, *p* < 0.01) than the DEF-fed rats.

### 3.8. HIF-1 and SIRT-3 Gene Expressions

**Figure 5:**
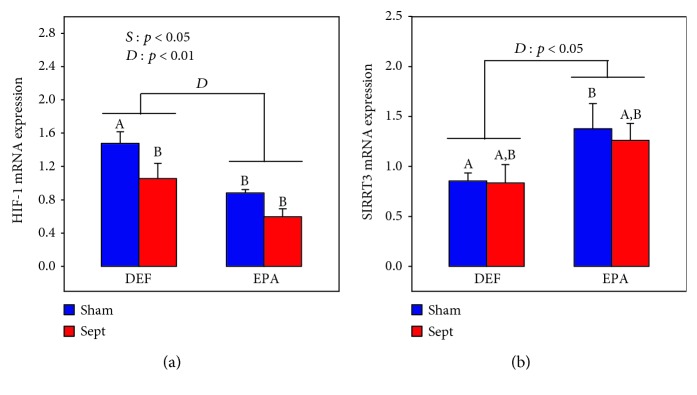
Myocardial hypoxia-inducible factor-1 (HIF-1) (a) and sirtuin-3 (SIRT3) (b) mRNA expressions. The means are the average of 8 rats. DEF: rats fed the *ω*3 polyunsaturated fatty acid-deficient diet; EPA: rats fed the eicosapentaenoic acid-enriched diet; Sham: sham-operated rats; Sept: septic rats; *D*: diet effect; *S*: sepsis effect; A, B: histogram bars on the same panel without a common letter are significantly different.

Reactive oxygen species (ROS) in the mitochondrial matrix are known to regulate the expressions of hypoxia-inducible factor-1 (HIF-1) and sirtuin-3 (SIRT3) [37]. Since mitochondria of the DEF group seemed to overproduce ROS, the gene expressions of these two proteins were thus evaluated in the whole myocardium. HIF-1 mRNA expression (Figure 5(a)) was affected by both the diet and the type of surgery. The presence of EPA in the diet strongly reduced the gene expression of this parameter (−40% and −44% for the Sham and Sept subgroups, *p* < 0.01). Sepsis had also a lowering effect (−29% and −33% for the DEF and EPA groups, *p* < 0.05).

SIRT3 mRNA expression (Figure 5(b)) evolved differently with an increasing effect of dietary EPA (+61 and +51% for the Sham and Sept subgroups, *p* < 0.05) and no significant influence of sepsis.

### 3.9. Mitochondrial Uncoupling Protein-3 (UCP3), Sirtuin-3 (SIRT3), and Myocardial Voltage-Dependent Anion Channel (VDAC) Expressions

**Figure 6:**
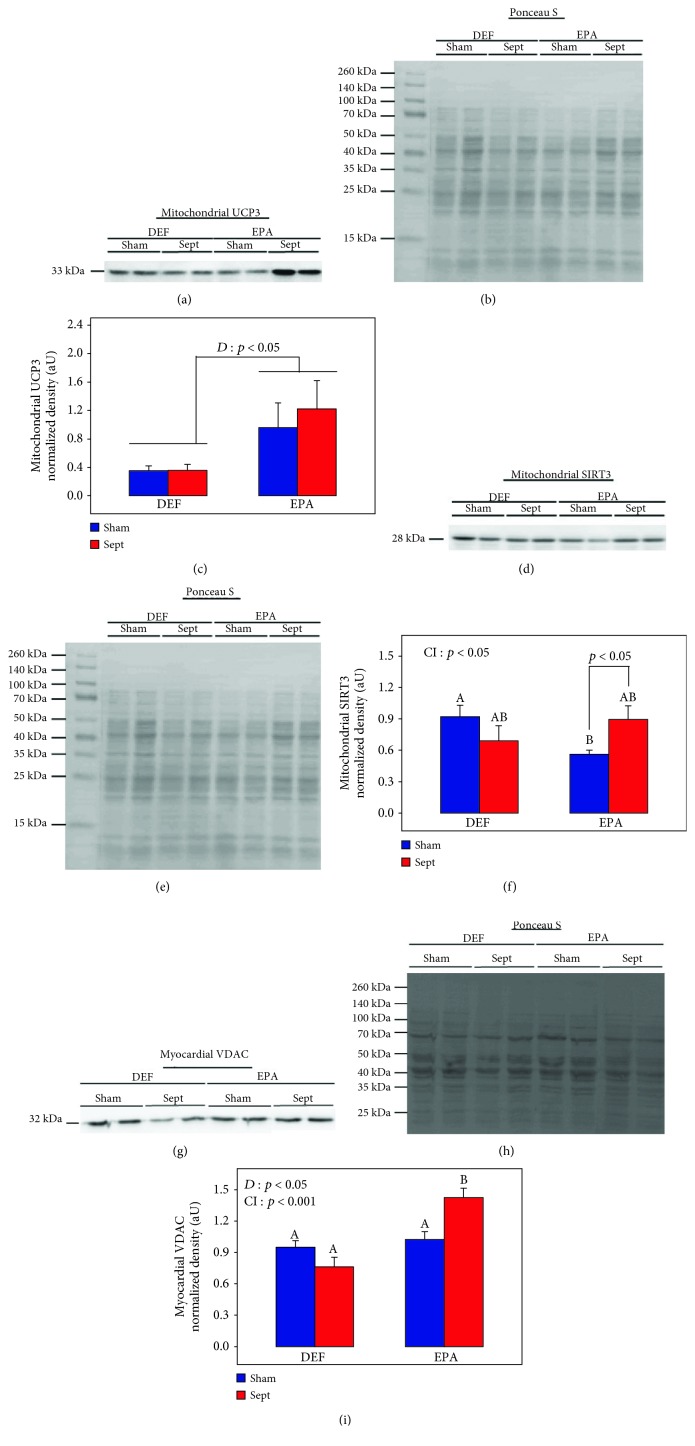
Mitochondrial uncoupling protein-3 (UCP3) (a–c), mitochondrial sirtuin-3 (SIRT3) (d–f), and myocardial voltage-dependent anion channel (VDAC) (g–i) expressions. (a, d, g) Representative immunoblots for UCP3, SIRT3, and VDAC; (b, e, h) protein densitometries to Ponceau S of the corresponding samples; (c, f, i) quantifications of mitochondrial UCP3 and SIRT3 together with myocardial VDAC. The means are the average of 8 rats. DEF: rats fed the *ω*3 polyunsaturated fatty acid-deficient diet; EPA: rats fed the eicosapentaenoic acid-enriched diet; Sham: sham-operated rats; Sept: septic rats; *D*: diet effect; CI: cross-interaction; A, B: histogram bars on the same panel without a common letter are significantly different.

UCP3 is known to modulate mitochondrial oxidative and nitrosative stresses [38].  Figure 6(c) shows UCP3 expression in the mitochondria. Dietary EPA greatly increased the expression of this protein irrespective of the surgery (+207%, *p* < 0.05). Sepsis did not influence the expression of this protein.

Mitochondrial sirtuin-3 protein expression (Figure 6(f)) was influenced by the diet and type of surgery. In the sham-operated subgroups, its content was lower when the diet was enriched with EPA (−39%, *p* < 0.05). Sepsis induced opposite effects on this parameter according to the dietary conditions (CI: *p* < 0.05). To examine this significant cross-interaction more closely, a one-way analysis of variance describing the effect of sepsis was performed in each dietary group: in the DEF group, the effect of sepsis was not significant (−25%, NS), but in the EPA group, sepsis significantly increased the amount of the protein (+60%, *p* < 0.05).

The myocardial content of mitochondria was evaluated by estimating the protein content of the mitochondrial voltage-dependent anion channel (VDAC) in the whole organ and isolated organelles. In the myocardial tissue (Figure 6(i)), VDAC was increased by the EPA-rich diet (+43% in general, *p* < 0.01). The increase in the Sham rats was low (+8%, NS) but was more marked in the CLP group (+87%, *p* < 0.01). The significant cross-interaction indicated that VDAC tended to be decreased by sepsis in the DEF group (−20%, NS) but increased by the pathology in the EPA group (+39%, *p* < 0.01).

In the isolated mitochondria, the VDAC content was not affected by the diets and the surgical procedures (data not shown).

Since all these results suggest an increased mitochondrial content in the septic myocardium of the EPA group, PGC-1*α* protein expression was determined in the different subgroups. The results indicated that the dietary and surgical interventions did not modify the expression of this protein (data not shown).

## 4. Discussion

This study set out to (i) determine whether the function of cardiac mitochondria is prematurely modified—before the occurrence of contractile dysfunctions—in early low-severity sepsis induced by cecal ligation and puncture in rats fed a Western diet, (ii) find out whether dietary eicosapentaenoic acid (C20:5 *n*-3 or EPA) protects the integrity of these organelles, and (iii) seek a potential mechanism responsible for observed effects. We found that the septic animals fed the Western-type diet displayed a reduced mitochondrial oxidative phosphorylation although the cardiac mechanical function was not yet reduced. This was associated with an increased mitochondrial oxidative stress. Dietary EPA exerts cardioprotection against these changes through reducing the mitochondrial oxidative stress, which may be related to increased mitochondrial UCP3 and SIRT3 expressions.

### 4.1. Justification of the Dietary Model

In the present study, the influence of sepsis was studied in two different populations of rats fed distinct dietary PUFAs. The first population was fed a diet in which the PUFAs were strictly *ω*6 (linoleic acid). This diet was thus deficient in *ω*3 PUFAs. It modeled some features of the Western diet, which is characterized by an excessive ingestion of *ω*6 PUFAs and an insufficient *ω*3 PUFA intake. In industrialized societies, the ratio of dietary *ω*6 PUFAs to *ω*3 PUFAs is around 15 but the World Health Organization recommends a ratio close to 4–5. EPA, one of the *ω*3 substrates of cyclooxygenase and lipoxygenase, was thus added to the second diet.

Dietary EPA decreased *ω*6 PUFAs in cardiac phospholipids, namely, arachidonic acid, C22:4*ω*6, and C22:5*ω*6, and increased long-chain *ω*3 PUFAs (C22:5 and C22:6*ω*3). These modifications are classically observed when a *ω*6 PUFA-based diet is enriched with *ω*3 PUFAs [17, 18, 39]. The PUFA (*ω*6 + *ω*3) level of phospholipids is relatively constant to maintain stability of the membrane fluidity. However, there is a perpetual renewal of acyl moieties in the constitution of these complex lipids. It is due to the activities of phospholipases A1 and A2 which deacylate the phospholipids. The resulting lysophospholipids are immediately reacylated by lysophospholipid acyltransferases which implant acyl moieties from acyl-CoA in these molecules. PUFAS of the *ω*6 series and those of the *ω*3 family compete for these enzymes. Introducing *ω*3 PUFAs in the diet thus induces a decrease in *ω*6 PUFAs and an increase in *ω*3 PUFAS in cardiac membranes.

### 4.2. Limitations of the Study

The maximal sepsis duration was 48 h and the intensity of the microbial infection was low (ligature at only 1 cm from the cecal apex). The sepsis was limited to this severity (i) for the welfare of the animals and to meet the requirements of the local ethics committee on the use and care of laboratory animals and those of the animal welfare cell of our laboratory and (ii) to limit animal death and so have a sufficient sample size to validate statistical analysis. Clearly, the results obtained are restricted to this short postsurgery period and low-severity sepsis and do not allow extrapolation to other situations.

### 4.3. Effects of the Diet and Surgical Procedure on Cardiac Mechanical Function

Cardiac mechanical function was estimated by MRI in the living animal anesthetized with isoflurane. The proportion of isoflurane was strictly regulated as a function of the respiratory rhythm. Body temperature of the animals (37°C) was also strictly controlled by adjusting the temperature of the gas flow entering the lungs.

Our model of sepsis had little effect on cardiac morphology and function. Sepsis has different influences on cardiac function according to the severity of the pathology: early sepsis increases the heart rate [6, 40] to fight against the systemic hypotension encountered in this situation [41]. With more severe sepsis, diastolic dysfunctions occur [8]. Finally, with evolution toward septic shock, systolic dysfunctions appear [7]. The early low-severity sepsis induced in our study did not trigger the expected increase in the heart rate occurring in the hyperdynamic phase of the pathology. However, the rate of contraction tended to be increased in both groups (+13%, *p* = 0.08), suggesting a compensatory mechanism for a probable hypotension. CLP in the rodent is known to decrease body temperature [42]. In our study, the body temperature was maintained at 37°C during the MRI measurements. Since we increased the temperature artificially, stimulation of the rate of contraction may have occurred instead of the increased heart rate.

### 4.4. Effects of the Diet and Surgical Procedure on Mitochondrial Function

In the sham-operated rats, the EPA-induced changes in the fatty acid composition of membrane phospholipids affected the cardiac mitochondria. Oxidative phosphorylation was unchanged, but several factors related to the oxidative stress were altered. Nitrosylation of mitochondrial proteins was decreased by EPA. This decrease suggests attenuation of reactive oxygen species (ROS) generation. Furthermore, the SOD2 mRNA expression tended to be decreased in this group (−40%, *p* = 0.13). SOD2 is the mitochondrial enzyme, and the lowering of its mRNA expression associated with the lower protein nitrosylation suggests that ROS impregnation of the mitochondrial matrix was reduced. The *ω*3 PUFA-related decrease in mitochondrial oxidative stress is of utmost importance in regulating the oxidative phosphorylation: ROS can block the Krebs cycle by inhibiting aconitase [43]. EPA-induced reduction of mitochondrial ROS impregnation can thus improve the oxidative phosphorylation. The phenomenon was associated with important changes in intermediary metabolism. Lysine acetylation reflecting the intensity of the *β*-oxidation rate [36] was decreased by the EPA diet, indicating a lower fatty acid oxidation. Furthermore, the pyruvate dehydrogenase kinase 4 mRNA expression was reduced by the *ω*3 PUFA. Since this enzyme inhibits pyruvate dehydrogenase, this finding suggests that the oxidative decarboxylation of pyruvate to acetyl-CoA in the mitochondrial matrix was accelerated. Finally, dietary EPA seemed to shift substrate oxidation toward a cardioprotective pattern characterized by high glucose oxidation and low fatty acid degradation [34, 35].

Sepsis strongly depressed the oxidative phosphorylation of cardiac mitochondria in the DEF-fed animals modeling the human Western diet, although degradation of cardiac mechanical function was not yet observed. Compensatory energy production might have occurred through anaerobic glycolysis. Abnormal mitochondrial activity was associated with increased oxidative stress. This was ascertained by the behavior of isolated mitochondria: they displayed an increased ratio of H_2_O_2_ production to oxidative phosphorylation regardless of the substrate used. This indicates that the ROS release was always higher at a similar rate of energy production. The mitochondrial oxidative stress encountered in the myocardium of these rats can explain the reduced oxidative phosphorylation: ROS and calcium are recognized effectors of the mitochondrial permeability transition pore (mPTP) [44]. The mPTP opening allows the release of small-size water-soluble entities (MW < 1500 Da) from the mitochondrial matrix, namely, NADH and adenine nucleotides, and this release reduces mitochondrial oxidative phosphorylation [17]. The sepsis-induced deficit in mitochondrial energy production translated into a reduction of mitochondrial protein acetylation reflecting a decreased *β*-oxidation rate. Pyruvate oxidation was not increased in compensation (unchanged glycolytic enzyme mRNA expressions). The mitochondrial oxidative stress also explains the augmentation of IL-1*β* mRNA expression in the myocardium: inflammatory cytokine expression is favored by mitochondrial ROS and can trigger a mitochondrial oxidative stress in a vicious cycle [45]. The cardiotoxicity after CLP was thus partly due to inflammation, which probably resulted from the accumulation of the proinflammatory *ω*6 PUFAs in membrane phospholipids [23, 46].

In the animals fed the EPA-rich diet, the oxidative phosphorylation of myocardial mitochondria was not depressed by sepsis. As ascertained by the lower SOD2 mRNA expression and ROS release by isolated mitochondria, the EPA-induced protection was due to a reduction of the matrix oxidative stress. This situation allowed (i) maintenance of the oxidative phosphorylation through prevention of mPTP opening, (ii) upholding of the intensity of the intermediate metabolism characterized by an increased *β*-oxidation rate despite an apparent reduction of glycolysis suggested by the decrease in gene expression of enzymes directly involved in the glycolytic pathway (HK2 and PKm), and (iii) a decrease in the inflammatory response characterized by a slackening of the NF-*κ*B pathway and reduction of IL-1*β* gene expression. This underlines the cardioprotective effects of EPA on sepsis, which is well documented in the literature by numerous human studies [47–62].

VDAC is essential to maintaining a high rate of oxidative phosphorylation, since the pore constituted by this protein allows the inward and outward movements of solutes through the outer mitochondrial membrane. Its expression in the whole myocardium was increased by dietary EPA in the septic subgroup, although it was unchanged in the mitochondria. This suggests an increased mitochondrial density. Yet PGC-1*α* was not overexpressed, indicating that mitochondrial biogenesis was unchanged: it is thus probable that mitophagy was slackened by the lower oxidative stress, leading to the progressive accumulation of nitrosylated proteins. In the long term, this might be detrimental for the heart and explain the few human studies describing a lack of EPA-induced cardioprotection [62–64] or even a negative effect [65, 66].

### 4.5. Mechanisms of the Cardioprotective Effects of Dietary EPA

#### 4.5.1. Dietary EPA in Sham-Operated Animals

Our study shows that dietary EPA increased mitochondrial UCP3 content. Such an observation was already noticed in C2C12 cells [67]. The reduced matrix oxidative stress of the EPA-enriched heart was triggered by the observed increase in mitochondrial UCP3 content [68]. UCP3 allows upholding of the oxidative metabolism [69]: UCP3 expression facilitates matrix expulsion of fatty acids and reduces their degradation in the *β*-oxidative pathway. Thus, a higher matrix-free CoA content is available for pyruvate oxidation. The assumed UCP3-related lower mitochondrial acyl-CoA content can also account for the reduced matrix oxidative stress accumulation of these toxic lipids in the mitochondria being known to favor ROS production [70]. The phenomenon was associated with increased SIRT3 and decreased HIF-1 mRNA expressions. Bell et al. [37] report in primary mouse embryo fibroblast that a decrease in the matrix oxidative stress induces a reduction of HIF-1*α* expression. EPA reduced the matrix oxidative stress and preconditioned the myocardium in terms of mRNA expressions. When the mitochondrial function was measured in the isolated organelles, no change in mitochondrial function was observed except an EPA-induced increase in the respiratory control ratio (RCR) when palmitoylcarnitine/malate was used as a substrate (+38%, *p* < 0.05). It is known that the UCP3-induced reduction of the oxidative stress is related to lipid oxidation: ROS open the mPTP and favor proton leakage [44], and this decreases the RCR. The EPA-related ROS scavenging activity improved the RCR, but the enhancement was limited to the reactive oxygen species resulting from the *β*-oxidative pathway. The changes in protein and/or gene expressions seen in the myocardium of sham-operated animals probably constitute preconditioning allowing the EPA-induced cardioprotection during sepsis.

#### 4.5.2. Dietary EPA in Septic Animals

The lower oxidative stress of the mitochondrial matrix of the EPA-fed rats was maintained during sepsis. It allowed (i) the reduction of inflammatory cytokine production, (ii) the maintenance of the mPTP in a closed configuration in the *in vivo* situation, and (iii) the upholding of high rates of oxidative phosphorylation to sustain the cardiac mechanical function.

In this model of early sepsis, UCP3 expression was still high in the cardiac mitochondria of animals treated with EPA. This was associated with an increased protein nitrosylation. An UCP3-induced reduction of oxidative stress in favor of an increased nitrosative stress has already been described under conditions of high ROS production [38]. The reduction of the oxidative stress prevented the mPTP opening and favored the upholding of the oxidative phosphorylation. Our study shows for the first time that the nitrosative stress is less deleterious for the mitochondria than the oxidative stress in these conditions of early sepsis. Furthermore, mitochondrial SIRT3 was also increased. Under stressing conditions, this NAD^+^-dependent deacetylase is translocated into the nucleus and mitochondria [71]. In the nucleus, the histone deacetylase has been reported to limit NF-*κ*B activation [72]. It thus limits the inflammation process [73] and related oxidative stress of the mitochondrial matrix. This effect may be potentiated by high expression of SIRT3 activating several enzymes involved in ROS scavenging activity [74]. Moreover, the increased protein acetylation observed in the septic mitochondria of the EPA group suggests stimulation of the *β*-oxidative pathway. Protein acetylation has strong regulatory effects on several mitochondrial enzymes [75]: it blocks glucose and fatty acid oxidations through inhibition of pyruvate, very long-chain acyl-CoA, and long-chain acyl-CoA dehydrogenases, but it stimulates enoyl-CoA hydratase/3-hydroxyacyl-CoA dehydrogenase [76], potentially leading to the accumulation of the amphiphilic and perhaps toxic 3-ketoacyl-CoA. By acting on a limited number of key enzymes, SIRT3 is able to overcome these deleterious effects via deacetylation of the involved proteins [77]: it thus allows the upholding of high rates of *β*-oxidation and oxidative phosphorylation.

### 4.6. Conclusion (See Figure 7)

Sepsis induces dysfunctions of cardiac mitochondria before contractile abnormalities in rats fed a Western-type diet. They are characterized by oxidative stress and disruption of oxidative phosphorylation. Dietary EPA and/or its elongation products (DPA and DHA) exert a strong cardioprotective effect, defined by the absence or delay of inflammation and upholding of the energy metabolism in the physiological range. The reason for the *ω*3 PUFA-induced anti-inflammatory effect is a low mitochondrial ROS impregnation suspected to be related to increased UCP3 and SIRT3 expressions. These effects already emerge in the physiological situation through changes in mRNA expressions that precondition the myocardium. They can explain the resistance to sepsis. The antioxidant effect of n-3 PUFAs has been regularly observed after short-term administration (until 10 weeks) of these fatty acids [78–80]. However, Firuzi et al. [81] noticed tendencies toward an increased circulating 8-iso-PGF2*α* concentration and decreased erythrocyte glutathione peroxidase and catalase activities after a 6-month treatment in patients enduring dilated cardiomyopathy. Further investigations are thus necessary to determine whether long-term administration of n-3 PUFAs has antioxidant and anti-inflammatory properties. Moreover, future studies with human cardiac mitochondria are now needed to confirm the protection observed in the rat during early sepsis.

## Figures and Tables

**Figure 7:**
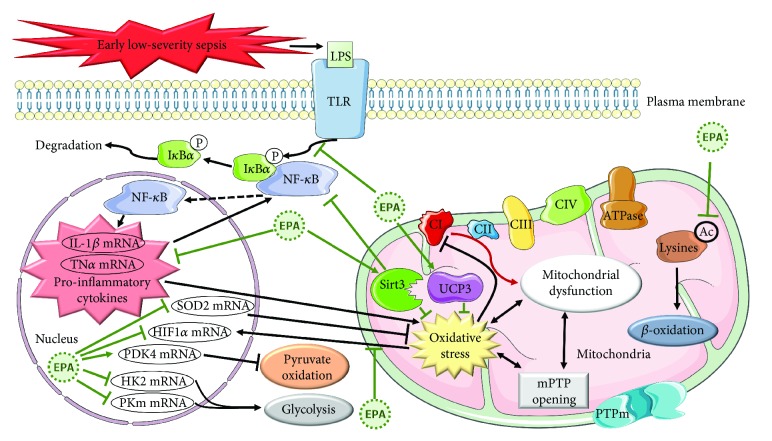
Effects of early low-severity sepsis and dietary eicosapentaenoic acid on myocardial function. LPS: lipopolysaccharides; TLR: Toll-like receptors; NF-*κ*B: nuclear factor-kappa B; I*κ*B*α*: nuclear factor of kappa light polypeptide gene enhancer in B-cell inhibitor alpha; P: phosphate; IL-1*β*: interleukin-1*β*; TNF-*α*: tumor necrosis factor-alpha; mRNA: messenger ribonucleic acid; EPA: eicosapentaenoic acid; HIF-1*α*: hypoxia-inducible factor-1-alpha; SOD2: mitochondrial superoxide dismutase; PDK4: pyruvate dehydrogenase kinase 4; HK2; hexokinase 2; PKm: pyruvate kinase; lysine acetylation: image of the beta-oxidation rate; SIRT3: sirtuin-3; UCP3: uncoupling protein 3; CI, CII, CIII, and CIV: complexes 1, 2, 3, and 4 of the respiratory chain; ATPase: adenosine triphosphate synthase; mPTP: mitochondrial permeability transition pore.

**Table 1:** Fatty acid composition of the diets (%).

	DEF	EPA
C14:0	0.1	0.1
C16:0	6.5	5.7
C18:0	2.7	3.0
C20:0	0.1	0.1
C24:0	0.1	0.1
SFAs	9.5	9.0
C16:1 *ω*7	0.1	0.1
C18:1 *ω*9	40.8	26.6
C18:1 *ω*7	0.9	0.8
C20:1 *ω*9	0.1	0.4
MUFAs	42.0	27.9
C18:2 *ω*6	47.1	44.7
C20:2 *ω*6	nd	0.2
C20:3 *ω*6	nd	0.1
C20:4 *ω*6	nd	0.8
*ω*6 PUFAs	48.1	46.9
C18:3 *ω*3	0.1	0.1
C20:4 *ω*3	nd	0.4
C20:5 *ω*3	nd	15.5
*ω*3 PUFAs	0.1	15.6
*ω*6/*ω*3	481	3

SFA: saturated fatty acid; MUFA: monounsaturated fatty acid; PUFA: polyunsaturated fatty acid; nd: not detected.

**Table 2:** Real-time PCR primers used in this study.

Gene	Sequence (5′-3′)
*β*-Actin	(F) TCTGTGTGGATTGGTGGCTCTA (R) CTGCTTGCTGATCCACATCTG
HIF-1	(F) AAGCACTAGACAAAGCTCACCTG (R) TTGACCATATCGCTGTCCAC
HK2	(F) GAGCTACCACGCACCCTAC (R) CACCCTTACTCGGAGCACAC
HPRT	(F) TTGCTGACCTGCTGGATTAC (R) AGTTGAGAGATCATCTCCAC
IL-1*β*	(F) CACCTTCTTTTCCTTCATCTTTG (R) GTCGTTGCTTGTCTCTCCTTGTA
LDHH	(F) ACCCCGTGTCTACAATGGTG (R) GGTTGATGACGCTGGTCAGT
LDHM	(F) TGAAGTCTCTGAACCCGCAG (R) CGTATGCACTGTCAACCACC
PDK4	(F) CACACCTTCACCACATGCTC (R) CGTGAATTGTCCATCACAGG
PFK2	(F) TTGCGAGGAAAGCAGTTTGC (R) ACACGCCAGCATCAATCTCA
PKm	(F) GTCAATCCACAGACCCCCTG (R) CCACTTGGTGAGCACTCCTG
SIRT3	(F) TGCTACTCATTCTTGGGACCTC (R) CTGTACCGATTCAGACAAGCTG
SOD1	(F) CAGGACCTCATTTTAATCCTCAC (R) TGCCCAGGTCTCCAACAT
SOD2	(F) CCGAGGAGAAGTACCACGAG (R) GCTTGATAGCCTCCAGCAAC
TNF-*α*	(F) GCCTCTTCTCATTCCTGCTC (R) GAGCCCATTTGGGAACTTCT

(F): forward; (R): reverse.

**Table 3:** Fatty acid composition of cardiac phospholipids.

Fatty acid	DEF Sham	DEF Sept	EPA Sham	EPA Sept
C14:0	0.13 ± 0.03	0.11 ± 0.02	0.12 ± 0.01	0.10 ± 0.01
C15:0	0.15 ± 0.02	0.14 ± 0.02	0.14 ± 0.01	0.13 ± 0.02
C16:0	10.3 ± 0.2^b^	10.2 ± 0.4^b^	10.7 ± 0.4^ab^	11.2 ± 0.1^a^
C17:0	0.18 ± 0.01	0.18 ± 0.01	0.18 ± 0.01	0.18 ± 0.01
C18:0	22.3 ± 0.2	22.4 ± 0.1	22.2 ± 0.3	21.9 ± 0.1
C20:0	0.16 ± 0.01	0.15 ± 0.01	0.16 ± 0.01	0.16 ± 0.01
C22:0	0.27 ± 0.01	0.28 ± 0.02	0.27 ± 0.02	0.26 ± 0.02
C24:0	0.13 ± 0.01	0.14 ± 0.01	0.14 ± 0.01	0.14 ± 0.01
SFA	33.7 ± 0.3	33.7 ± 0.4	34.0 ± 0.2	34.2 ± 0.2
C16:1 *ω*7	0.20 ± 0.01	0.23 ± 0.02	0.21 ± 0.02	0.23 ± 0.01
C18:1 *ω*9	4.1 ± 0.2^a^	3.7 ± 0.1^a^	3.2 ± 0.1^b^	3.1 ± 0.2^b^
C18:1 *ω*7	2.8 ± 0.1	2.9 ± 0.1	2.6 ± 0.1	2.6 ± 0.1
*trans*-MUFA	0.18 ± 0.01	0.19 ± 0.01	0.20 ± 0.01	0.19 ± 0.01
C22:1 *ω*9	0.3 ± 0.1	0.2 ± 0.1	0.3 ± 0.1	0.2 ± 0.1
MUFA	7.6 ± 0.1^a^	7.3 ± 0.2^a^	6.7 ± 0.3^b^	6.4 ± 0.2^b^
C18:2 *ω*6	17.6 ± 0.5^ab^	17.4 ± 0.6^a^	19.0 ± 0.3^b^	18.5 ± 0.6^ab^
C20:2 *ω*6	0.18 ± 0.01	0.17 ± 0.01	0.16 ± 0.01	0.16 ± 0.01
C20:3 *ω*6	0.25 ± 0.01^a^	0.27 ± 0.01^a^	0.32 ± 0.01^b^	0.32 ± 0.01^b^
C20:4 *ω*6	22.5 ± 0.5^a^	23.3 ± 0.5^a^	18.6 ± 0.8^b^	17.3 ± 0.3^b^
C22:4 *ω*6	1.86 ± 0.08^a^	1.68 ± 0.05^b^	0.29 ± 0.02^c^	0.33 ± 0.02^c^
C22:5 *ω*6	5.1 ± 0.3^a^	4.6 ± 0.2^b^	0.2 ± 0.1^c^	0.1 ± 0.1^c^
*ω*6 PUFA	47.4 ± 0.6^a^	47.3 ± 0.7^a^	38.6 ± 0.8^b^	36.7 ± 0.4^b^
C18:4 *ω*3	0.08 ± 0.02^a^	0.14 ± 0.01^b^	0.11 ± 0.01^ab^	0.12 ± 0.02^ab^
C20:5 *ω*3	nd^a^	nd^a^	0.79 ± 0.08^b^	0.77 ± 0.04^b^
C22:5 *ω*3	0.7 ± 0.1^a^	0.7 ± 0.1^a^	4.3 ± 0.3^b^	4.6 ± 0.1^b^
C22:6 *ω*3	6.6 ± 0.6^a^	6.6 ± 0.4^a^	11.4 ± 0.4^b^	12.9 ± 0.6^c^
*ω*3 PUFA	7.4 ± 0.6^a^	7.5 ± 0.4^a^	16.6 ± 0.6^b^	18.4 ± 0.5^c^
Total PUFA	54.8 ± 0.2	54.9 ± 0.4	55.2 ± 0.4	55.1 ± 0.3
*ω*6/*ω*3	6.6 ± 0.7^a^	6.4 ± 0.4^a^	2.4 ± 0.1^b^	2.0 ± 0.1^b^

The means correspond to the average of 5 rats. DEF Sham: rats fed the *ω*3 fatty acid-deficient diet with fictitious surgery; DEF Sept: rats fed the *ω*3 fatty acid-deficient diet with cecal ligation and puncture; EPA Sham: rats fed the EPA-rich diet with fictitious surgery; EPA Sept: rats fed the EPA-rich diet with cecal ligation and puncture; SFA: saturated fatty acids, MUFA: monounsaturated fatty acids; PUFA: polyunsaturated fatty acids; nd: not detected; a, b: means without a common letter on each line are significantly different.

**Table 4:** *In vivo* cardiac morphology and function.

	DEF Sham	DEF Sept	EPA Sham	EPA Sept	ANOVA
EDV	298 ± 26	326 ± 20	347 ± 27	306 ± 20	
ESV	92 ± 8	113 ± 8	119 ± 15	95 ± 9	CI: *p* < 0.05
SEV	194 ± 16	231 ± 13	228 ± 16	211 ± 14	
EF	65 ± 2	65 ± 4	66 ± 3	69 ± 2	
HR	371 ± 6	366 ± 12	355 ± 7	374 ± 8	
CO	229 ± 6	217 ± 24	227 ± 7	236 ± 11	
CR	0.22 ± 0.02	0.25 ± 0.01	0.23 ± 0.02	0.26 ± 0.02	*S*: *p* = 0.085
RR	0.18 ± 0.01	0.17 ± 0.01	0.19 ± 0.02	0.19 ± 0.02	

The means represent the average of 8 rats. DEF Sham: rats fed the *ω*3 fatty acid-deficient diet with fictitious surgery; DEF Sept: rats fed the *ω*3 fatty acid-deficient diet with cecal ligation and puncture; EPA Sham: rats fed the EPA-rich diet with fictitious surgery; EPA Sept: rats fed the EPA-rich diet with cecal ligation and puncture; EDV: end-diastolic volume (*μ*l); ESV: end-systolic volume (*μ*l); SEV: systolic ejection volume (*μ*l/beat); EF: ejection fraction (%); HR: heart rate (beats/min); CO: cardiac output (ml/min/g of wet weight); CR: contraction rate (cm^2^/s); RR: rate of relaxation (cm^2^/s); ANOVA: analysis of variance. The two-way analysis of variance describes the effects of the diets, those of sepsis (*S*), and the cross-interaction between these two factors (CI).

**Table 5:** Mitochondrial function.

Substrate	Parameter	DEF Sham	DEF Sept	EPA Sham	EPA Sept
GM	State 3	84 ± 7^a^	49 ± 7^b^	67 ± 5^ab^	87 ± 6^a^
	State 4	13 ± 1^a^	10 ± 1^a^	11 ± 1^ab^	15 ± 1^a^
	RCR	6.5 ± 0.2^a^	4.5 ± 0.5^b^	6.3 ± 0.2^a^	6.0 ± 0.1^a^
	ATP	379 ± 49^a^	239 ± 58^b^	411 ± 47^a^	416 ± 35^a^
	ATP/O	2.3 ± 0.2	2.6 ± 0.6	3.1 ± 0.3	2.4 ± 0.2
	ROS state 3	81 ± 5	87 ± 7	79 ± 9	80 ± 7
	ROS state 4	113 ± 7	123 ± 12	126 ± 18	119 ± 14
	ROS/state 3	1.0 ± 0.1^a^	1.7 ± 0.2^b^	1.1 ± 0.2^a^	0.9 ± 0.1^a^
	ROS/state 4	9.7 ± 0.9^ab^	12.6 ± 2.1^a^	10.8 ± 0.8^ab^	8.4 ± 1.0^b^
	ROS/ATP	0.25 ± 0.04^a^	0.57 ± 0.18^b^	0.21 ± 0.03^a^	0.20 ± 0.03^a^

SR	State 3	129 ± 13	101 ± 13	120 ± 12	137 ± 16
	State 4	52 ± 3	46 ± 4	49 ± 3	46 ± 2
	RCR	2.5 ± 0.1	2.2 ± 0.1	2.5 ± 0.1	2.7 ± 0.2
	ATP	293 ± 42^ab^	208 ± 33^b^	321 ± 21^a^	290 ± 19^ab^
	ATP/O	1.1 ± 0.1	1.1 ± 0.1	1.3 ± 0.1	1.2 ± 0.1
	ROS state 3	139 ± 6	146 ± 6	140 ± 6	143 ± 8
	ROS state 4	174 ± 7	175 ± 11	173 ± 10	181 ± 15
	ROS/state 3	1.2 ± 0.1^a^	1.6 ± 0.2^b^	1.2 ± 0.1^a^	1.1 ± 0.1^a^
	ROS/state 4	3.4 ± 0.2	3.9 ± 0.3	3.4 ± 0.1	3.3 ± 0.1
	ROS/ATP	0.60 ± 0.13^ab^	0.82 ± 0.13^a^	0.44 ± 0.03^b^	0.48 ± 0.02^b^

PM	State 3	111 ± 10	93 ± 14	124 ± 14	129 ± 15
	State 4	12 ± 1	11 ± 2	10 ± 1	11 ± 1
	RCR	9.1 ± 0.4^a^	8.6 ± 0.9^a^	13 ± 1^b^	12 ± 1^b^
	ATP	616 ± 69	507 ± 103	621 ± 68	656 ± 46
	ATP/O	2.6 ± 0.2	2.6 ± 0.3	2.6 ± 0.35	2.73 ± 0.2
	ROS state 3	56 ± 5	57 ± 7	64 ± 11	59 ± 8
	ROS state 4	90 ± 9	88 ± 11	98 ± 17	82 ± 8
	ROS/state 3	0.5 ± 0.1^a^	0.7 ± 0.1^b^	0.5 ± 0.1^a^	0.5 ± 0.1^a^
	ROS/state 4	7.6 ± 0.8	8.3 ± 1.0	9.8 ± 1.2	8.2 ± 0.9
	ROS/ATP	0.10 ± 0.02^ab^	0.14 ± 0.03^c^	0.10 ± 0.01^ac^	0.09 ± 0.01^ab^

The means correspond to the average of 8 animals. DEF Sham: animals fed the *ω*3 fatty acid-deficient diet with the fictitious surgery; DEF Sept: animals fed the *ω*3 fatty acid-deficient diet with the cecal ligation and puncture; EPA Sham: animals fed the EPA-rich diet with the fictitious surgery; EPA Sept: animals fed the EPA-rich diet with the cecal ligation and puncture; GM: glutamate/malate; SR: succinate/rotenone; PM: palmitoylcarnitine/malate; state 3: state 3 respiration rate (nmoles O_2_/min/mg of proteins); state 4: state 4 respiration rate (nmoles O_2_/min/mg of proteins); RCR: respiratory control ratio; ATP: rate of ATP production (nmoles of ATP/min/mg of proteins); ATP : O: ratio between the production of ATP and the state 3 respiration rate (nmoles of ATP/nmoles of O_2_); ROS state 3 and ROS state 4: H_2_O_2_ release during the state 3 and state 4 respirations (pmoles/min/mg of proteins); ROS/state 3: ratio between H_2_O_2_ release during state 3 and oxygen consumption during state 3 (pmoles of H_2_O_2_/nmoles of O_2_); ROS/state 4: ratio between H_2_O_2_ release during state 4 and oxygen consumption during state 4 (pmoles of H_2_O_2_/nmoles of O_2_); ROS/ATP: ratio between H_2_O_2_ release during state 3 and rate of ATP production (pmoles of H_2_O_2_/nmole of ATP); a, b, c: means without a common letter for the same parameter are significantly different.

**Table 6:** Glycolytic enzyme mRNA expressions in the myocardium.

	DEF Sham	DEF Sept	EPA Sham	EPA Sept	ANOVA
HK2	1.20 ± 0.12^ab^	1.69 ± 0.22^a^	1.03 ± 0.15^ab^	0.52 ± 0.05^b^	*D*: *p* < 0.001 CI: *p* < 0.01
PFK2	2.36 ± 0.28^a^	1.97 ± 0.22^ab^	2.35 ± 0.27^a^	1.38 ± 0.24^b^	*S*: *p* < 0.05
PKm	0.94 ± 0.07^a^	0.80 ± 0.11^ab^	0.66 ± 0.10^b^	0.26 ± 0.03^c^	*D*: *p* < 0.001 *S*: *p* < 0.01
LDHM	0.91 ± 0.02^a^	0.98 ± 0.16^a^	0.87 ± 0.07^a^	0.55 ± 0.05^b^	*D*: *p* < 0.05
LDHH	1.54 ± 0.16^ab^	1.76 ± 0.30^b^	1.10 ± 0.19^a^	0.44 ± 0.08^c^	*D*: *p* < 0.001 CI: *p* < 0.05
PDK4	0.68 ± 0.14^a^	0.52 ± 0.07^ac^	0.26 ± 0.06^bc^	0.06 ± 0.01^b^	*D*: *p* < 0.001

The means represent the average of 8 rats. DEF Sham: rats fed the *ω*3 fatty acid-deficient diet with fictitious surgery; DEF Sept: rats fed the *ω*3 fatty acid-deficient diet with cecal ligation and puncture; EPA Sham: rats fed the EPA-rich diet with fictitious surgery; EPA Sept: animals fed the EPA-rich diet with the cecal ligation and puncture; HK2: hexokinase 2; PFK2: phosphofructokinase 2 inhibiting the phosphofructokinase 1; PKm: pyruvate kinase muscle type; LDHM: muscular lactate dehydrogenase catalyzing the transformation of pyruvate to lactate; LDHH: cardiac lactate dehydrogenase catalyzing the transformation of lactate to pyruvate; PDK4: pyruvate dehydrogenase kinase 4 inhibiting the pyruvate dehydrogenase by phosphorylation; ANOVA: analysis of variance. The two-way analysis of variance describes the effects of the diets (*D*), those of sepsis (*S*), and the cross-interaction between these two factors (CI); a, b, c: means without a common letter for the same parameter are significantly different.

## Data Availability

The datasets generated during and/or analysed during the current study are available from the corresponding author on reasonable request.

## Abbreviations

ArA: Arachidonic acid
ADETEC: Surgical association for the development and enhancement of the techniques of screening and treatment of cardiovascular diseases
ADP: Adenosine diphosphate
AIDS: Acute immunodeficiency syndrome
ANOVA: Analysis of variance
ARRIVE: Animal research: reporting of *in vivo* experiments
ATP: Adenosine triphosphate
CaCl_2_: Calcium chloride
cDNA: Complementary deoxyribonucleic acid
CoA: Free coenzyme A
CO_2_: Carbon dioxide
Cu-Zn: Copper-zinc
DEF: *ω*3 polyunsaturated fatty acid-deficient diet
DHA: Docosahexaenoic acid
DICOM: Digital imaging and communications in medicine
ΔΨ: Mitochondrial membrane potential
*dP*/*dt*_max_: Maximal rate of rise in the left ventricular-developed pressure
*dP*/*dt*_min_: Maximal rate of decrease in the left ventricular-developed pressure
ECG: Electrocardiogram
ECL: Enhanced chemiluminescence
EDTA: Ethylenediaminetetraacetic acid
EDV: End-diastolic volume
EGTA: Ethylene glycol-bis(2-aminoethylether)-*N*,*N*,*N*′,*N*′-tetraacetic acid
EPA: Eicosapentaenoic acid
ESV: End-systolic volume
FADH_2_: Reduced flavin adenine dinucleotide
FFC: French Federation of Cardiology
FoxO3a: Forkhead box O3 transcription factor
FRAP: Ferric reducing antioxidant power
HCl: Hydrochloric acid
HEPES: 4-(2-Hydroxyethyl)-1-piperazineethanesulfonic acid
HIF-1: Hypoxia-inducible factor-1
H_2_O_2_: Hydrogen peroxide
HRP: Horseradish peroxidase
HSE: Hugo Sachs Elektronik
IL-1*β*: Interleukin-1*β*
KCl: Potassium chloride
KH_2_PO_4_: Potassium dihydrogen phosphate
LA: Linoleic acid
LSD: Least significant difference
LV: Left ventricular
MgSO_4_: Magnesium sulfate
MOPS: 3-(*N*-Morpholino)propanesulfonic acid
mPTP: Mitochondrial permeability transition pore
MR: Magnetic resonance
MRI: Magnetic resonance imaging
mRNA: Messenger ribonucleic acid
MUFA: Monounsaturated fatty acid
NaCl: Sodium chloride
MW: Molecular weight
NADH: Reduced nicotinamide adenine nucleotide
NaHCO_3_: Sodium bicarbonate
NF-*κ*B: Nuclear factor kappa-B
NCSS: Number cruncher statistical software
O_2_: Oxygen dioxide
*ω*: Omega
PUFA: Polyunsaturated fatty acid
PVDF: Polyvinylidene difluoride
qRT-PCR: Quantitative real-time polymerase chain reaction
RCR: Respiratory control ratio
RNA: Ribonucleic acid
ROS: Reactive oxygen species
Sept: Septic
SDS-PAGE: Variant of polyacrylamide gel electrophoresis
SEM: Standard error of the mean
SIRT3: Sirtuin-3
SV: Systolic volume
SOD: Superoxide dismutase
TBARS: Thiobarbituric acid-reactive substances
TNF-*α*: Tumor necrosis factor-alpha
UCP3: Uncoupling protein-3
VDAC: Voltage-dependent anion-selective channel.
